# Cryopreservation and Transfer of Sheep Embryos Recovered at Different Stages of Development and Cryopreserved Using Different Techniques

**DOI:** 10.3390/ani13142361

**Published:** 2023-07-20

**Authors:** Marina I. Selionova, Magomet M. Aibazov, Ekaterina K. Zharkova

**Affiliations:** 1 Subdepartment of Animal Breeding, Genetics and Biotechnology, Russian State Agrarian University—Moscow Timiryazev Agricultural Academy, Timiryazevskaya Street, 41, 127343 Moscow, Russia; m_selin@mail.ru; 2 North Caucasian Agrarian Center, Zootechnicheski 15, 355017 Stavropol, Russia; velikii-1@yandex.ru

**Keywords:** Charollais ewes, donors, superovulation, embryos, slow freezing, vitrification, recipients, transfer, survival rate

## Abstract

**Simple Summary:**

Due to gene pool insufficiency, it is clear to use the best breeds of sheep for meat. Multiple Ovulation and Embryotransfer Technology (MOET) is applied to improve the genetic and production performance of high-value ewes. This article presents cryoresistance data for Charollais sheep embryos, considering the stage of embryo development and the freezing method. No significant differences in survival rate and morphological structure between cryopreserved and fresh embryos were found. But, cryoresistance largely depended on embryonic development stages. Thus, cryopreservation of embryos at the 6 days after insemination was more effective than freezing 2–3-day embryos after insemination. The data obtained is believed to make assisted reproductive technologies (ART) more effective and cheaper. This article will be interesting for farm producers, breeders and researchers.

**Abstract:**

This article presents data from experiments to determine the cryoresistance of Charollais sheep embryos, depending on the stage of embryo development and the method of freezing, as well as the results of embryo transfer. The study design consisted of a study on the cryopreservation of ewe embryos at different developmental stages (early, 2–8 blastomeric and late, at the morula/blastocyst stage), two cryopreservation protocols (slow freezing and ultra-fast vitrification), and embryo transfer of cryo- and fresh embryos. Embryos from Charollais sheep donors (n = 12) were recovered after induction of superovulation. The embryos were recovered surgically (laparotomy) on days 2 and 6 after insemination. Before there was transfer to recipients, part of embryos was cryopreserved using standard slow freezing and ultra-fast vitrification methods. The average ovarian response was 7.54 ovulations per donor, and 5.83 embryos per donor were collected. No effect of the cryopreservation method and embryo development stage on the preservation of the morphological structure of embryos was found. There were no significant differences in the survival rate of cryoembryos at different development stages, frozen using different techniques, and after transfer to recipients. Differences in cryoresistance between embryonic developmental stages in favor of the morula/blastocyst stage were found (survival after thawing 86.4% vs. 75.0% in early embryos). At different stages of development, the survival rate of fresh embryos (45.8%) compared to cryopreserved ones (30.2%) was significantly higher (*p* < 0.05), while among fresh ones, the best survival rate (50.0%) was observed after the transfer of morules and blastocysts.

## 1. Introduction

In the last half century, assisted reproductive technologies (ART) have been used to improve the genetic and production performance of farm animals [1]. Artificial insemination (AI) has become the most widespread among the many developed ART, which allows the maximum use of high-value producers and has been implemented in animal husbandry in many countries, including Russia [2]. Other ART, including Multiple Ovulation and Embryotransfer Technology (MOET) [3], aimed at the efficient use of high-value females and has been widely applied in cattle breeding and in small ruminants, in some countries with developed sheep breeding [4,5]. In Russia, although the MOET technology is fairly well developed, it is used only for scientific purposes and it is not used in practical sheep breeding, primarily because of its low efficiency [6].

It is known that for biotechnology to be of interest to agricultural producers, it must be effective and inexpensive. Researchers are unanimous in the opinion that the effectiveness of MOET depends on many factors, which is confirmed by rather contradictory data obtained by different authors. Under the same conditions, embryos at the blastocyst stage have been reported to be more viable in vivo compared to embryos at the late morula stage [7], and embryo survival rate was dependent on the stage of embryo development and was higher with blastocyst transfer compared to zygote transfer at an earlier stage [8].

Some researchers [9,10] found that the survival rate of blastocysts freshly recovered and frozen by vitrification did not differ significantly. Moreover, there are reports of the effect of the freezing method and embryo stage on embryo transfer performance [11]. For example, studies have shown that transferring embryos that were vitrified at the morula stage resulted in a higher pregnancy rate than transferring embryos at the morula stage that were cryopreserved by slow freezing [12]. It has been observed that embryos cryopreserved using the slow freezing method had a higher rate of embryo fragmentation than vitrified embryos [13].

There are no studies on the use of MOET using cryoembryos in sheep husbandry practice in Russia. Moreover, we found no data in the national scientific literature on the results of cryopreservation and vitrification of sheep embryos at different developmental stages. The experiment purpose was to study, in the Charollais breed sheep, the effect of the stage of embryo development and the method of cryopreservation on pregnancy rate and embryo survival rate after transfer to recipients. Thus, apparently, our experiments are among the first studies on the stimulation of multiple ovulations and the effectiveness of cryopreservation and embryo transplantation in meat sheep farming in Russia.

## 2. Materials and Methods

The present study was conducted from September 2021 (breeding season) to March 2022 on experimental farm of the All-Russian Research Institute of Sheep and Goat Breeding, Stavropol, Russia. All experimental animals were housed in a covered shelter where there was an open-air pen and a grazing area. The ration included alfalfa hay, barley and mixed concentrate according to recommendations. Water, salt and minerals were available ad libitum. The authors state that all procedures in the experiment were carried out in accordance with the principles of animal welfare; the animals were cared for and handled by qualified personnel.

### 2.1. Study Design

Twelve adult Charollais ewes (body weight: 58.5 ± 4.0 kg) with a positive birth history were used as embryo donors. The ewes were clinically healthy and checked by ultrasonography (USG, portable ultrasound scanner EDAN DUS 60 VET) for absence of pregnancy and internal genital abnormalities. Sheep were examined transcutaneously using a convex abdominal probe and transrectally using a rectal linear probe. The ultrasound scanner was set to real-time B-mode (2D) and 5.0 MHz. The superovulatory induction programme was carried out as follows: a polyurethane intravaginal sponge impregnated with 45 mg of fluorogeston acetate (Muqimuye Sci-Tech Co., Ltd., Shanghai, China) was administered to each donor ewe (day of application—day 0). Simultaneously with the pessary insertion, 125 µg of prostaglandin F2α (PGF2α; Estrofan, Bioveta, Ivanovice na Hane, Czech Republic) was injected subcutaneously. On day 12, from the start of treatment, follicle-stimulating hormone (Folltropin-V, Bioniche Animal Health, Belleville, ON, Canada) was injected, 12 h apart, at a dose of 2.5 and 2.0 mL, respectively. On day 13, injection dose was 1.5 + 1.5 mL, on day 14, it was 1.0 + 1.0 mL, on day 15, a single dose of 0.5 mL was given. 36 h after pessary removal, the donor ewes were intramuscularly injected with 200 IU of human chorionic gonadotropin (hCG, Chorulon^®^, Animal Health, Inc., Madison, NJ, USA). On Day 15 (12 h after sponge withdrawal), estrus detection with vasectomized rams. All ewes showing signs of behavioral estrus were artificially inseminated intracervically three times with fresh semen (200 × 10^6^ viable spermatozoa in 100 μL). The interval between inseminations was 8 h.

Embryos were collected surgically by laparotomy either on day 2 or day 6 after insemination depending on the desired embryonic stage. Intravenous injection of 0.3–0.4 mL of Telasol (Telasol, Zoetis, Parsippany, NJ, USA) was used as a sedative for general anesthesia. Dulbecco’s solution (DPBS, Gibgo, Billings, MT, USA) was used to flush the uterine horns to collect embryos in sterile EmSafe filter systems (MiniTube, Tiefenbach, Germany).

### 2.2. Cryopreservation of Embryos

Slow freezing was performed in medium-containing ethylene glycol and sucrose according to the method described by Martinez and Matkovic, 1998 [14]. The straws with embryos were placed in a programmable freezer (IMV, L’Aigle, France) at temperature of 25 °C, then were cooled at a rate of 2 °C/min and incubated at minus 6 °C for 5 min. Then, the embryos were cooled at a rate of 0.3 °C/min to minus 33 °C. After this step of the freezing process, straws were placed in a liquid nitrogen thermos at −196 °C for storage.

For vitrification, embryos were consecutively placed at 25 °C in three solutions containing increasing concentrations of ethylene glycol (10%, 20% and 40%) in the base medium, and then placed in a fourth medium containing 0.125 M trehalose with 10% FBS. The embryos were transferred into mini straws, which were immersed directly into liquid nitrogen.

### 2.3. Thawing Procedures

Thawing was carried out according to the technique proposed by [15], which involved thawing the embryos after a slow freeze for 20 s in a water bath at 37 °C, while the thawing of straws with vitrified embryos was performed at room temperature with further washing of defrosted embryos in washing solutions to remove ethylene glycol. All procedures were performed at room temperature (25 °C). Morulae were immersed for 5 min in a BM solution containing half the concentration of the cryoprotectants present in the final solution of vitrification: BM +20 EG% + 0.5 M sucrose. Then, all morulae were transferred in BM + 0.5 M sucrose and BM + 0.25 M sucrose, for 5 min each. Finally, morulae were transferred to two BM solutions for 2.5 min each. The thawed embryos were evaluated under inverted microscope (Olympus, Tokyo, Japan) and ensured that the embryos recovered their morphology. Depending on the morphology, we divided them into four categories: (1) morphologically normal embryos; (2) injured embryos: these embryos had several degenerative changes in the inner cell mass area with dark and necrotic cells; (3) lysed cells: ruptured embryo after freezing and thawing procedure and (4) damaged shiny membrane: loss of *zona pellucida*. Only embryos classified as high quality (code 1: excellent/good or 2: satisfactory; IETS classification [16] were selected for transfer.

### 2.4. Embryo Transfer

Edilbay ewes were used as recipients. A total of 72 recipients were divided into six groups. The experimental groups (Groups 1–4, 48 ewes in total, 12 ewes per group) consisted of ewes depending on the embryo cryopreservation procedures used and the different stages of embryonic development. Control groups (groups 5–6, 24 ewes in total and 12 ewes per group) of ewes to which fresh embryos were transferred: group 5—transfer of 2–8 cell stages; group 6—transfer of embryos at the morula/blastocyst stage. The estrous period was synchronized in all recipients using the following procedure. After intravaginal injection of a sponge impregnated with 45 mg fluorogestone acetate (Muqimuye Sci-Tech Co., Ltd., Shanghai, China) (day of application = day 0). On day 9, the intravaginal sponges were removed and 350 IU of gonadotropin (PMSG; Folligon^®^, MSD Animal Health, Inc., Madison, NJ, USA) was administered to all ewe recipients. The time gap between the estrous cycles of donors and recipients did not exceed 12 h. This condition was observed for ewes of all groups. The presence and number of *corpora lutea* (*CL*) in the recipients were observed using laparoscopy. Embryos were transferred laparoscopically (2 embryos per recipient, regardless of the number of *CL*) into the oviduct (transfer of 2–8 cell stages on day 2 after estrus onset) and into the uterine horn (transfer of morula/blastocyst stages on day 6 after estrus onset). Embryos were transferred into one oviduct or uterine horn ipsilateral to the ovaries with *CL*. In cases with double ovulation in different ovaries, embryos were transferred to both oviducts or horns.

### 2.5. Diagnosis of Pregnancy

Post-embryontransfer pregnancy was diagnosed at 35 days after embryo transfer by transrectal ultrasonography an EDAN DUS 60 VET (EDAN, Shenzhen, China) using a 5.0 MHz linear probe. The pregnancy rate in each group preliminary was expressed in % and was based on the number of pregnant ewes/number of recipients. All recipients were monitored until lambing when the number of offspring, sex and body weight of lambs at birth, 1, 2 and 4 months were recorded. Embryo survival rate was calculated by dividing the number of live lambs obtained by the number of embryos transferred, multiplied by 100 and expressed as a percentage.

### 2.6. Statistical Analysis

Parametric data (*CL* of donors and recipient ewes and body weight of lambs) were analyzed using a one-way ANOVA. For binomial data (proportion of normal, degenerated, lysed, damaged *zona pellucida* of embryos for slow freezing and vitrification, pregnancy rate at 35 days and embryo survival rate), contingency tables were analyzed using the Fisher exact test to determine statistical differences. A *p*-value of less than 0.05 was understood to indicate statistical significance.

## 3. Results

All donor ewes (n = 12) responded with multiple ovulation, as indicated by the presence of good morphology and color *CL* in the ovaries. No significant differences were found in the level of ovulations depending on the right or left ovary (48 and 42 *CL*, respectively).

The ovaries of the first group of donors (n = 6) which were operated on day 2 after insemination showed 47 *CL* (average of 7.9 ± 1.12 *CL* per donor). The number of recovered zygotes was 36 (76.6%). No unfertilized oocytes were found. Six embryos (16.6%) were retrieved at the 2 blastomer stage, 22 embryos (61.2%) at the 4 blastomer stage and 8 embryos (22.2%) at the 8 blastomer stage. Accordingly, 6.0 ± 1.21 normal zygotes were recovered per donor, which is an acceptable result.

The ovaries of the second group of donors (n = 6), operated on day 6 after insemination, showed only 43 *CL* (average of 7.17 ± 1.32 *CL* per donor). A total of 34 embryos were recovered (79.1%), including 12 embryos at the morula stage (35.3%) and 22 embryos at the blastocyst stage (64.7%). As in the first donor group, no unfertilized oocytes were detected. The average number of recovered embryos per donor in this group was 5.67 ± 1.10, which also corresponds to good outcome parameters. There were no significant differences between the groups in terms of extraction time, the number of yellow bodies or the number of embryos.

For cryopreservation, 20 zygotes at stages 2–8 blastomers and 22 embryos at the morula and blastocyst stages were selected. According to the experimental design, within each stage of embryo development, cryopreservation was carried out either by slow freezing or using a vitrification procedure. After cryopreservation, the embryos were thawed and their quality was determined. These results were presented in Table 1.

We found that most embryos at stage 2–8 blastomeres cryopreserved by slow freezing (n = 10) had normal morphology after thawing (code 1–2 (good and satisfactory)—8 cells (80.0%); code 3 (degenerated)—1 cell or 10.0%; code 4 (lysed)—1 cell or 10.0%).

After thawing embryos at developmental stages 2–8 blastomeres cryopreserved using vitrification procedures (n = 10), most zygotes were also morphologically normal (code 1–2, 7 cells or 70.0%; code 3–4, degenerated and with a damaged pellucid zone, 3 embryos or 30.0%).

In the group of 11 embryos slow-frozen at the morula/blastocyst stage, after thawing there were 9 embryos with normal morphology (code 1–2, 81.8%); 2 embryos with a damaged pellucid zone (18.2%). Embryos vitrified at the morula/blastocyst stage (n = 11) after thawing had the following quality parameters: with normal morphology (code 1–2, a total of 10 cells (90.9%); damaged—1 cell (9.1%).

As a result of hormonal induction, the total number of *CL* in all recipients was 112, with an average of 1.55 ± 0.076 per ewe: 57 *CL* was detected in the right ovary (mean number was 0.79 ± 0.076) and 55 *CL* was in the left (mean number was 0.76 ± 0.061). This indicates that there are no significant differences between the ovulatory response of the ovaries (*p* > 0.05). The ultrasound diagnostics revealed no significant difference in the pregnancy rate between groups 1–4 (mean 47.9% with a limit of 41.7–50.0%). There were no differences in pregnancy rates between the 5 and 6 groups—an average of 70.85% with a limit of 66.7% and 75.0%. Summing up the data on the stages of development in the groups of frozen and fresh embryos showed that at the stage of 2–8 cells, embryo survival rate occurred in 26.3%, at the stage of morula/blastocyst—29.1% (*p* > 0.05). Thus, there was no significant difference in the frequency of pregnancy depending on the stage of the embryo, both frozen and fresh (Table 2). A comparison of freezing techniques also revealed no differences in the onset of pregnancy after embryo transplantation. With slow freezing, pregnancy occurred in 31.2%, whereas with vitrification, 29.16% (*p* > 0.05). Comparison of the survival rate of frozen and fresh embryos revealed significant differences by ultrasound test both in the number of pregnancies (35.4% vs. 23.9%, *p* ≤ 0.05) and in the number of lambs received (45.8% vs. 30.2%, *p* < 0.05).

There were no differences in the number of lambs of different sexes depending on the stage of embryo development and the method of freezing. Data on weight at birth and lamb weight at 1, 2 and 4 months of age are shown in Table 2. There was no difference in the live weight of the offspring at these time points, neither depending on the stage of development at which the embryos were collected from donors, nor on the method of freezing (*p* > 0.05). The live weight of the lambs obtained in groups 5 and 6 did not differ significantly in all time points between the stage of embryo development.

## 4. Discussion

Sheep and goat breeding are the most dynamic livestock industries in many countries of the world [17,18,19]. With many positive parameters of small ruminant breeding, there are biological limitations of intensifying reproduction in natural reproduction methods, such as the presence of sexual season, low fecundity, getting 1 lamb per year, etc. The use of assisted reproductive technologies (ART), especially artificial insemination, having significantly increased the rate of breeding, creating many highly productive types and breeds of small ruminants [20]. With regard to the intensification of the use of females, this issue remains unresolved and only a limited number of countries widely use MOET technology in the breeding of small ruminants considering the specifics of the technology [21,22,23].

MOET is not widespread in Russia; however, in our opinion, this is not a reason to abandon scientific development in order to improve its individual elements, increase its efficiency and reduce its costs. The superovulatory response of Charollais donor ewes to the applied hormonal treatment scheme, ranged from 5 to 13 *CL*, the average for donor embryos (days 2 and 6) was 7.53, and transferable embryos—5.84. This can be explained by Charollais features, the effective hormonal treatment scheme and, perhaps, the authors’ practical experience in similar procedures.

The results of the present study show that the efficiency of superovulation, as determined by the number of *CL* and the number of embryos at the corresponding stage of development can be considered satisfactory. The number of *CL* ranged from 5 to 13 units, the average embryos for donors (days 2 and 6) was 7.53, and transferable embryos—5.84. These results were similar to the results of other researchers [24] and even slightly exceeded them [25]. Dias et al. used superovulation protocol that included an intravaginal device (0.36 g of progesterone) for nine days plus six decreasing doses of pFSH (333 IU, from 60 h before intravaginal device removal) and two doses of 37.5µg d-cloprostenol at the time of last two pFSH doses. The effectiveness of the protocol was in the range of 7.0–8.6 in the number of *CL*, and the collected embryos—3.7–6.2 per donor [24]. Figueira et al. to induce superovulation in donors used acetate medroxyprogesterone intravaginal sponges for six or nine days plus d-cloprostenol and eCG 24 h before sponge removal and conclude that the nine-day protocol promoted higher ovulation rate and embryo yield [25].

The fact that all the extracted embryos were fertilized suggests at least two important points. Firstly, the hormonal treatment scheme proved to be optimal, and biologically compatible with the natural physiology of reproduction in sheep, which provided the basis for the release of complete oocytes. Secondly, the triple artificial insemination of ewes with good-quality semen ensured a high level of ovum fertilization.

The mean number of recovered embryos at both stages of embryonic development compared to the number of observed *CL* can be explained by several factors. Perhaps the fimbriae of the oviduct were not able to completely cover the markedly enlarged ovaries resulting from the induction of superovulation and, therefore, oocytes were probably able to enter the abdominal cavity. When retrieving embryos at the morula / blastocyst stage, their losses are possible, but despite this, we recommend obtaining embryos from donor ewes on day six after insemination, as this is a simpler procedure.

In our previous study, when superovulation was induced in Charollais and Ile-de-France breeds, a washout was carried out on day six after insemination and similar results were obtained [6]. Garcia-Garcia et al. [11,26] and Shirazi et al. [27] revealed that the increase in cryotolerance from 2–4-cell embryos to the morula stage follows a linear manner and from the morula to the blastocyst stage in a quadratic manner. Frozen early cleavage stage embryos had a significantly lower viability than their fresh counterparts (23.1 vs. 83.1%; *p* < 0.0001, in one of the experiments [28] and 23.1% versus 66.1%, *p* < 0.0001 in another [11]), with a similar rate of viability between fresh or frozen blastocysts (92.5 vs. 83.7%). The number of dead cells in vitrified blastocysts was higher than for non-vitrified blastocysts (*p* < 0.05) [27].

Cryopreservation is known to reduce embryo quality [28,29,30,31]. In our experiments, cryopreservation, regardless of the stage of embryo development and freezing technology, damaged embryos to some extent. In each of the four groups, there were damaged embryos, including degenerated embryos with lysed cell morphology and embryos with a damaged pellucid zone. Damage to the latter is thought to result from mechanical stress, resulting in reduced viability of the blastomers [32,33].

The preservation of normal morphology in 70–90% of defrosted embryos found in our experiments is a good result and corresponds to the average values obtained by other researchers. It is noteworthy that the greatest damage (30% of the total number) was after the thawing of embryos at the developmental stage of 2–8 blastomers, cryopreserved using vitrification. After slow freezing, almost equal numbers of damaged and unsuitable for transfer embryos were observed in both 2–8 blastomeric zygotes (20%) and in morula/blastocyst stages (around 18%).

Embryos vitrified at the morula/blastocyst stage showed the best cryopreservation rates—90.9% of embryos had normal morphology after thawing. The explanation seems to lie in the fact that vitrification uses high concentrations of cryoprotectant, combined with fast cooling rates, to dehydrate the embryos. The prevention of ice crystal formation during freezing and thawing periods is necessary [34,35]. It is also possible that it is at this stage of development that sheep embryos are most resistant to external influences [31], but this assumption must be substantiated scientifically. Experiments involving cow [36] and goat [37,38] embryos have shown that the survival of vitrified embryos can be related to several factors, such as the use of different cryoprotectants, exposure time or concentration of vitrification solutions or different stages of embryonic development. Researchers cite several protocols and technical options to improve embryo survival after cryopreservation in goats [39] and sheep [40,41].

In the present study, the pregnancy rate in recipient ewes when transferring 2–8 cell embryos after slow freezing was the lowest at 41.7%. This result correlates with data [9,10], which reported that the pregnancy rate was higher with the transfer of vitrified blastocysts compared with the transfer of embryos frozen at the early stage. The pregnancy rate in recipient ewes when transferring 2–8 cell vitrified embryos and morulae/blastocysts frozen using both techniques was 50.0%, similar to data obtained by Green R.E. et al. [12], but significantly lower (60%) than in the. Dattena M. et al. [42] study. Thus, Green R.E. et al. no significant difference had observed among fresh, frozen or vitrified embryos on pregnancy rate (50.0%, 38.6% and 55.8%). However, when they evaluated only the direct transference, the pregnancy rate of open-pulled straw (OPS) vitrified embryos was higher than that of frozen early embryo stages (57.1% vs. 34.8%) (*p* = 0.07). In addition, vitrified morulae had a higher pregnancy rate than the ones with frozen embryos (64.0% vs. 38.9%) (*p* = 0.07) [12]. When using different concentrations and combinations of cryoprotectors (glycerol with ethylene glycol and dimethyl sulphoxide) survival rate varied in the 57.1–62.9% range [42]. The average pregnancy rate from embryo transfer usually correlates with the quality of frozen-thawed embryos, although in some studies, the pregnancy rate depends on the synchrony of the reproductive cycle of the donor recipient [43] or the completeness of the *CL* in recipient animals [44].

Pregnancy rate after the transfer of cryopreserved embryos survival rates did not differ significantly between groups 1–4 (range 29.8% to 33.3%), which correlates with the results of [43], in which 49 lambs (30.6%) were obtained from 80 recipients, in which 160 embryos were transferred. The highest survival rate (50.0%) was observed after the transfer of freshly obtained morula/blastocysts (group 6). The number of lambs, fetal birth weights and lambs’ weight at 1, 2 and 4 months of age did not differ significantly between groups 1–6. This result is in discordance with the results of the study [43], in which lamb body weight at two months of age was higher in lambs obtained using vitrification procedures compared to slow freezing procedures.

Thus, the results of the present study, using both traditionally accepted slow freezing and ultra-fast vitrification for embryo cryopreservation show that embryo quality was good with both techniques. The results of our study are consistent with those obtained by other researchers [45,46], in which the quality of frozen-thawed embryos was higher with vitrification procedures than with the slow-freeze method [47,48]. The advantages of using vitrification for embryo cryopreservation are simplicity, rapidity of the procedure and the fact that this method does not require expensive equipment as in the case of the slow freezing method.

## 5. Conclusions

The results of the experiment showed that the average ovarian response after the applied hormonal scheme of superovulation induction was 7.54 ovulations per donor, and 5.83 embryos per donor were collected. Differences in the cryoresistance observed at different stages of embryo development in favor of the morula/blastocyst stage (survival after thawing of embryos frozen at a late stage was 86.4% versus 75.0% in early embryos). There were no significant differences in the survival rate of embryos frozen using different technologies (slow freezing or vitrification) after transfer to recipients, while the survival rate of fresh embryos was significantly higher (45.8% vs. 30.2%). The collection of embryos at the morula/blastocyst stage (day six after insemination) and their cryopreservation by vitrification seems to be the most promising approach, but larger scale experiments are necessary to establish its possible superiority.

## Figures and Tables

**Table 1:** Post thawing quality of embryos collected on day 2 or day 6 after insemination and cryopreserved by slow freezing or vitrification.

Stage of Embryos	Freezing Method	Quality of Frozen-Thawed Embryos							
		Normal		Degenerated		Lysed		Damaged *zona pellucida*	
		n	%	n	%	n	%	n	%
2–8 cell embryos	slow freezing	8/10	80.0	1/10	10.0	1/10	10.0	-	-
	vitrification	7/10	70.0	-	-	-	-	3/10	30.0
Morula/ blastocyst stage	slow freezing	9/11	81.8	-	-	-	-	2/11	18.2
	vitrification	10/11	90.9	-	-	-	-	1/11	9.1

**Table 2:** Pregnancy rate and lambing rate in recipient ewes receiving either fresh or cryopreserved (slow freezing or vitrification) embryos (collected on day 2 or day 6 after insemination), embryo survival rate and embryo growth.

		Mean ± SEM					
		Stage of Frozen-Thawed Embryos				Stage of Fresh Embryos	
		2−8 Cells		Morulae and Blastocysts		2−8 Cells	Morulae and Blastocysts
		Slow Freezing	Vitrification	Slow Freezing	Vitrification		
		Group 1	Group 2	Group 3	Group 4	Group 5	Group 6
Number of recipients		12	12	12	12	12	12
Number of embryos transferred		24	24	24	24	24	24
Pregnancy rate at 35 days	number	5	6	6	6	8	9
	%	41.7	50.0	50.0	50.0	66.7	75.0
Number of offspring		7	7	8	7	10	12
Sex of lambs, ♂/♀		3/4	4/3	5/3	3/4	4/6	6/6
Embryo survival rate, %		29.17	29.17	33.33	29.17	41.67	50.00
Body weight of lambs, kg	at birth	3.08 ± 0.44	2.72 ± 0.35	3.15 ± 0.49	2.91 ± 0.51	3.12 ± 0.67	2.93 ± 0.55
	1 months	9.12 ± 0.96	8.88 ± 1.04	9.42 ± 1.12	8.36 ± 0.90	9.56 ± 1.15	8.74 ± 1.41
	2 months	17.67 ± 1.44	18.56 ± 1.56	18.98 ± 1.72	17.79 ± 1.88	19.12 ± 1.38	18.56 ± 1.77
	4 months	33.43 ± 2.12	32.56 ± 2.31	34.40 ± 2.89	33.76 ± 2.41	35.23 ± 2.57	34.87 ± 2.39

## Data Availability

Research data were presented in the tables in the main text of the article.
